# New archeological marvels of ancient Shu civilization

**DOI:** 10.1093/nsr/nwab071

**Published:** 2021-05-07

**Authors:** Weijie Zhao

**Affiliations:** NSR news editor based, Beijing

## Abstract

China Central Television broadcasted live reports on the latest archaeological excavation at the Sanxingdui site over four consecutive days, from 20–23 March 2021. Exquisite large golden mask fragments, as well as numerous bronze, jade, pottery and ivory artefacts, were unearthed in six newly discovered pits at the site. Following the public presentation of these ancient cultural relics, along with the contemporary archaeological excavation technologies, the mysterious Sanxingdui civilization has become a hot topic within the Chinese media and scientific community.

## The discovery of Sanxingdui

The archaeological site at Sanxingdui (三星堆, or ‘three star mound’) is located on the southern bank of the Yazi River in the Sichuan Basin of south-west China. The Sanxingdui site covers an area of ∼12 square kilometres and is identified as being the capital city of the ancient Shu kingdom (古蜀国) ∼3600–3100 years ago, prior to the relocation of the capital to Jinsha (金沙), also in the Sichuan Basin.

**Figure 1:**
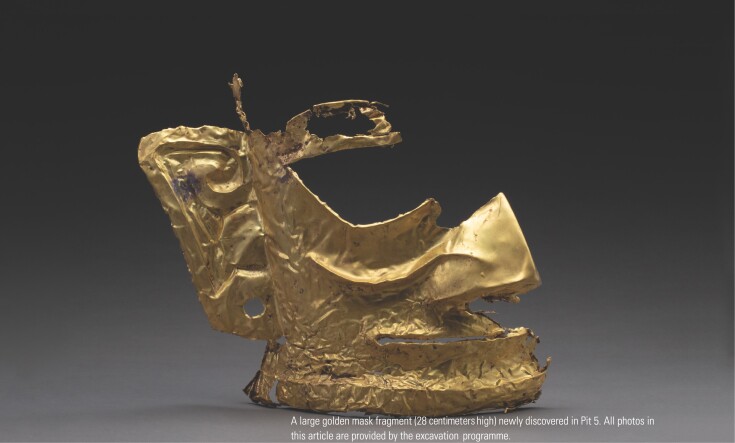


The Sanxingdui site was first discovered in 1929, and its most famous cultural relics were all excavated in 1986 from two adjacent pits in the southern part of the site. The dazzling relics include a bronze statue of a towering man (2.62 metres tall), a bronze mask with protruding eyes and big ears resembling the famous Shu King Yufu (鱼凫) (1.38 metres wide), bronze trees (one of which is 3.95 metres high) and a finely engraved gold sceptre (1.42 metres long).

The first two pits were accidentally discovered by local residents during soil collection for making bricks. Rescue excavations by archaeologists unearthed more than 1000 cultural relics, segregated in four layers that contained ivory, clay, gold/bronze and jade artefacts, respectively. Interestingly, most of these artefacts were deliberately burned and fragmented before burial.

It is unclear why these artefacts were buried. Were they used for sacrificial activities, or were they hastily broken and buried during the war prior to translocation to the new capital? The excavated items are unique, with styles not seen in ancient cultural relics found in central China. They raised the question of the origin of Shu culture and its role in Chinese civilization—an issue that remains unsolved.

During the renovation of the Sanxingdui Museum in November 2019, a new pit of artefacts was discovered close to Pit 2, right below the museum’s exhibition platform. The new pit's orientation and size, and the artefact layered pattern, are nearly identical to those of Pit 2. Five additional pits were subsequently found in the vicinity, making a total of eight pits at the Sanxingdui site.

With the completion of the newly constructed archaeological excavation shelter, a state-of-the-art multi-disciplinary, multi-institutional Sanxingdui archaeological excavation programme was launched on 6 September 2020. This programme is led by the Cultural Relics and Archaeology Research Institute (CRARI) of Sichuan Province, and involves collaborative work among 34 universities and institutions. By March 2021, Pits 3, 4, 5 and 6 had been excavated to the artefact layer, and more than 500 relics had been unearthed, including golden mask fragments, a bird-shaped gold foil, masks, trees and containers in bronze, as well as jade, pottery and ivory artefacts. Excavation of Pits 7 and 8 is ongoing.

‘In the past, we were unsure about the nature of these pits—whether they were for sacrificial, discarding, or burial purposes. The six new pits provided many new clues, and the sacrificial characteristics became increasingly obvious,’ said Tang Fei (唐飞; Chinese name; Tang is the family name), the dean of Sichuan CRARI and the head of the Sanxingdui excavation programme. ‘The artefacts in these pits were deposited in the same layering order, thus were unlikely to have been disposed in a state of panic before escaping. Moreover, the quality of these artefacts is very high, suggesting their association with kings or high-level nobilities. Thus, the pits were probably created during well-organized, large-scale sacrificial ceremonies.’

Nevertheless, it is difficult to ascertain the sacrificial scene in the ancient Shu kingdom three millennium ago. Wang Wei (王巍), President of the Chinese Archaeological Society, who leads the advisory group for the Sanxingdui excavation programme, noted that ‘large-scale sacrificial activities should be carried out in temples or other sacrificial places rather than in these pits. Where is the temple? Was it destroyed? Can we find the ruins? These are open questions.’

## Technology-assisted excavation

The Sanxingdui archaeological excavation has been ongoing since September 2020, with a staff of 120 working concurrently at the site. The excavation will last for at least one or two years. ‘Excavation is a complex work. These artefacts are extremely fragile and it is very difficult to unearth them,’ said Tang.

The greenhouse-like archaeological excavation shelter was constructed directly over the newly discovered pits. This enables the maintenance of a constant temperature and humidity over the excavation area, facilitating complex excavation and protecting the cultural relics. All staff wear protective clothing within the excavation areas to minimize pollution and avoid damaging the relics. On-site workshops and laboratories were established in the shelter for specialized work on relic protection, archaeological research, emergency diagnosis, organic and inorganic chemical analysis, and micro-marking analysis, which aim at immediate on-site solution of problems encountered during the excavation.

**Figure 2:**
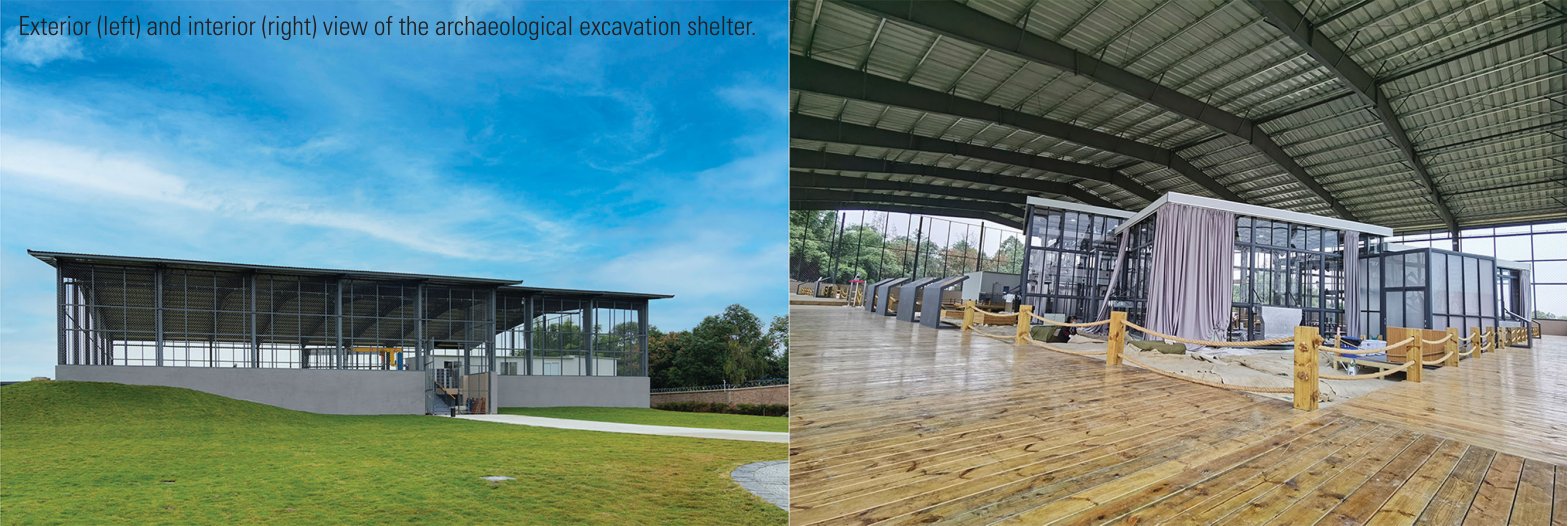


**Figure 3:**
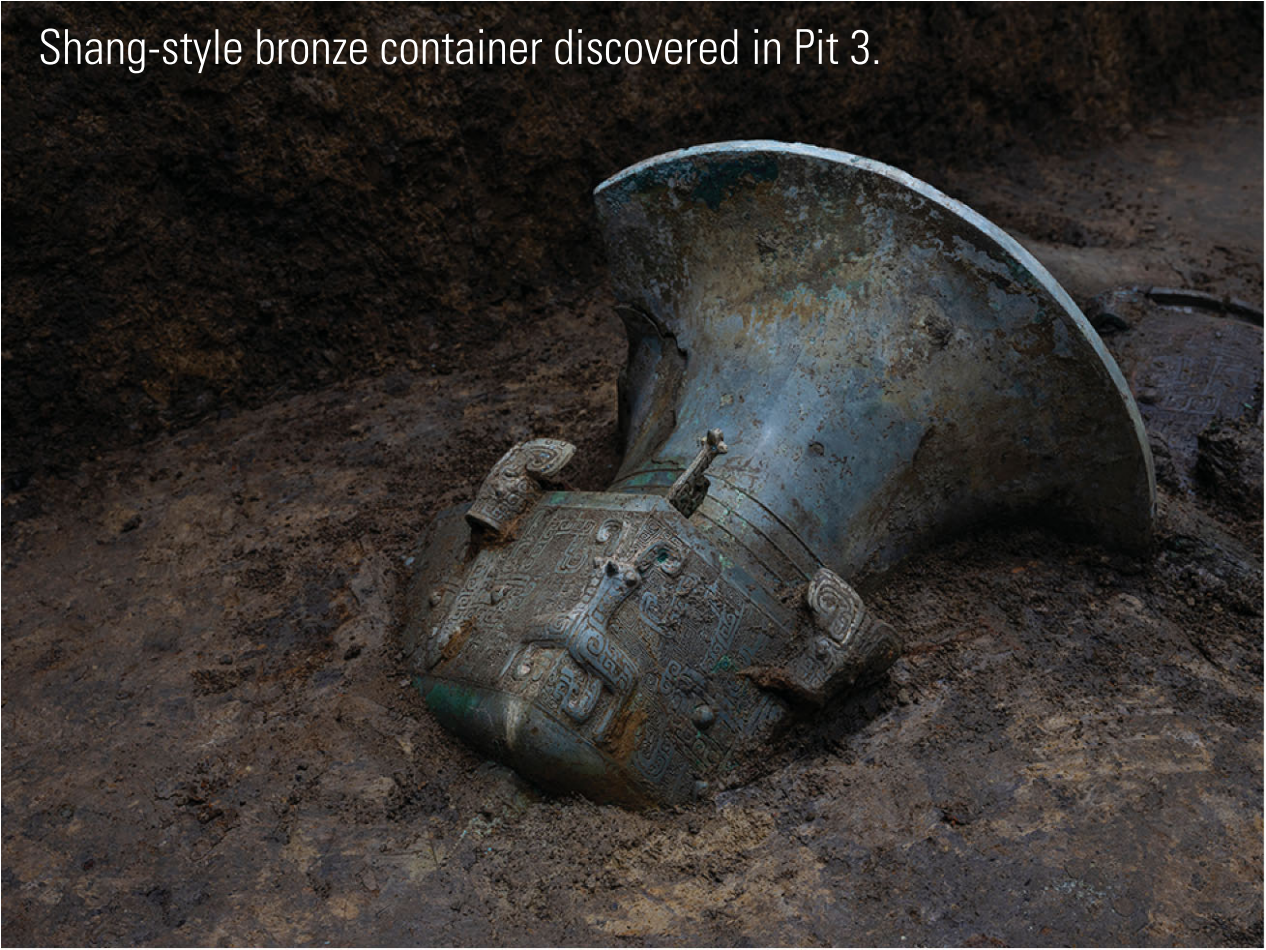


**Figure 4:**
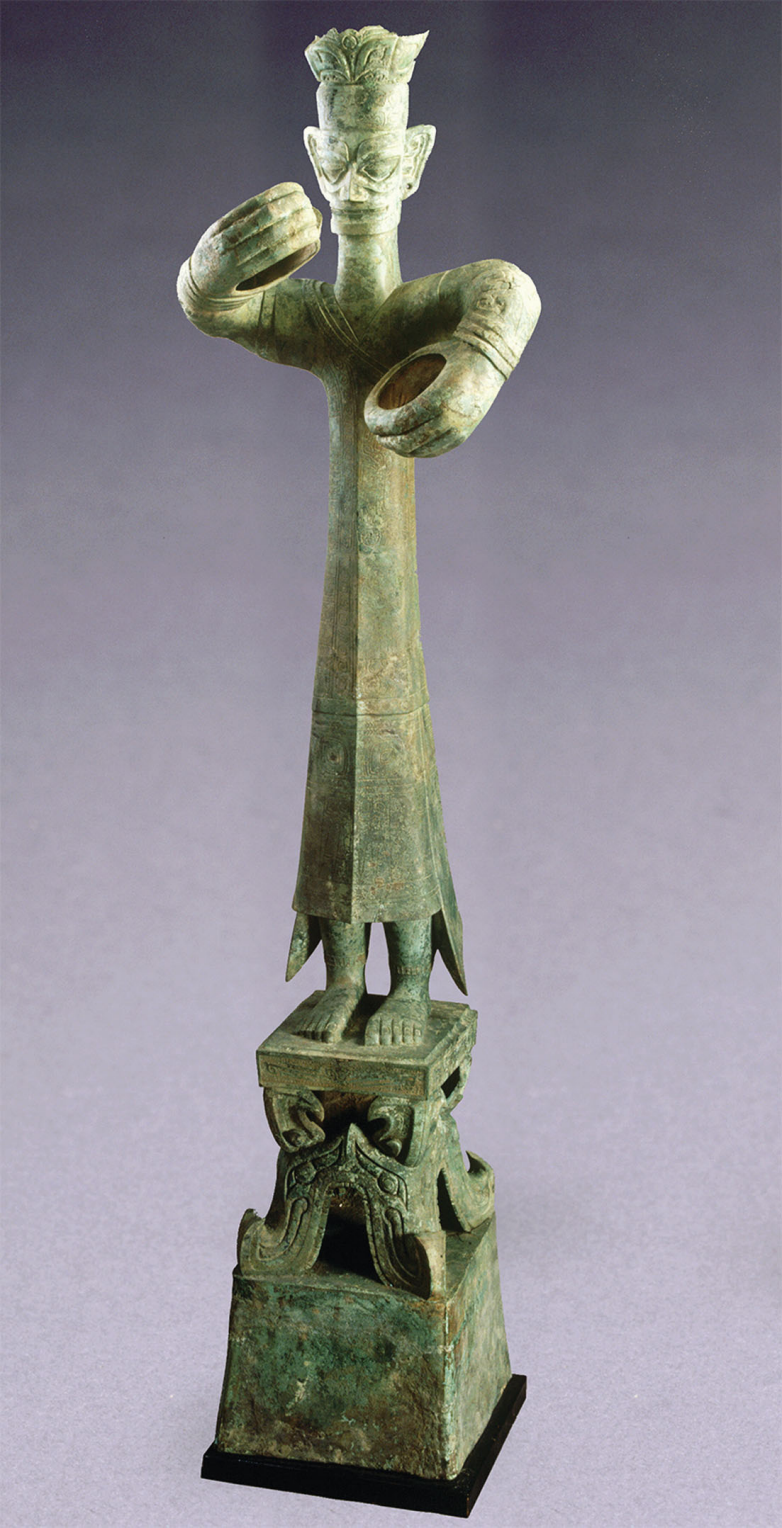
The 2.62-metres-tall bronze statue of a towering man, unearthed from Pit 2.

In addition to traditional technologies for physical exploration, spectroscopic analysis, plant archaeology and carbon-14 dating, the current project includes two new technologies that are a first in archaeological excavation—the 3D printing of tailored silicone protective cases for protecting individual relics during the unearthing process, and hyperspectral analysis for identification of unique features of each relic in order to establish a database.

Even with the application of all these technologies, the excavation work remains arduous. Tang explained: ‘The state of each relic is unique, so we have to formulate an appropriate workplan for each one. For example, there are hundreds of ivory in the pits. Although they look complete, they are as fragile as sand sculpture on the beach and nearly impossible to unearth without causing damage. The same goes for bronzes and other relics, since their internal structure may have been changed.’

In addition to the onsite work, many samples are sent to various research institutions for further analysis. For example, a team headed by Professor Wu Xiaohong (吴小红) at the School of Archaeology and Museology at Peking University has received more than 100 samples from the Sanxingdui site for radiocarbon dating analysis. This method can trace back 50 000–60 000 years and reach a precision of 5‰.

Most of the artefacts unearthed at Sanxingdui sites had been burned, so the organic matter is poorly preserved, causing difficulties in dating work. ‘Most samples are mixtures of charcoal detritus, bone residues, and soil, requiring purification of suitable carbon-containing components. The bamboo charcoal detritus in Pit 4 was relatively compact in structure, thus radiocarbon dating for Pit 4 went relatively smoothly, yielding a date between 1199 and 1017 BC,' said Wu. This corresponds to the late period of the Shang (商) dynasty in central China, and is consistent with previous dating of bronze and jade artefacts from Pits 1 and 2 based on type-morphology analysis.

The application of modern technologies has greatly facilitated the excavation. Nevertheless, as Tang stated, ‘Technologies are just auxiliary means. They offer useful clues but cannot determine what we can find and what new knowledge we can learn.’

Jing Zhichun (荆志淳), a professor of anthropology at the University of British Columbia in Canada, who did not participate in this excavation, agreed: ‘Multi-disciplinary archaeology requires not only various kinds of equipment, but, more importantly, advances in archaeological theories, and the integration of natural sciences with humanities and social sciences, especially anthropology, sociology, and art history. Only by this integration, can we truly understand the ancient Sanxingdui culture from the new discoveries, the entanglement of people and objects at that time, and the role of Sanxingdui culture in early Chinese civilization.’

## The multiplex early Chinese civilization

Another mystery of Sanxingdui is the uniqueness of its cultural relics. According to Wang, the distinguishing feature of the ancient Shu civilization is its belief system. The bronze trees, statues and masks all stem from their beliefs. On the other hand, many other unearthed relics also exhibit the deep influence of the central Chinese civilization. For example, many Sanxingdui jade and bronze artefacts are very similar to those unearthed in central China and are in line with the central Chinese etiquette system. Moreover, as Wang said, ‘it is obvious that the bronze metallurgy technology of Sanxingdui came from central China’.

The University of Science and Technology Beijing, the Palace Museum and the Sichuan CRARI are collaborating in an effort to splice and repair the ‘Bronze Tree 3’ from Pit 2, aiming to reconstruct a complete bronze tree from over 70 fragments.

According to Chen Kunlong (陈坤龙), a professor at the University of Science and Technology Beijing and an expert in archaeometallurgy, the alloy recipes and piece mould casting process used for making Bronze Tree 3 belong to the same technical tradition as used in the Central Plain and other regional bronze civilizations in the Shang period. This reflects the close interaction between Sanxingdui and other contemporary cultures, despite the distinctive characteristics of Sanxingdui bronze objects.

‘The period right before Sanxingdui pits were created happened to be the most prosperous period of the Shang dynasty in central China,’ commented Wang, and further noted, ‘At that time, the Shang dynasty repeatedly attacked and strongly impacted the surrounding kingdoms.’ According to the recorded history, there were many smaller kingdoms surrounding the Shang dynasty, one of which was the Shu kingdom. Archaeologists have discovered the ruins of many ancient kingdoms throughout China, spanning the upper, middle and lower reaches of the Yangtze and Yellow Rivers. Even the ancient kingdom Sushen (肃慎), located in China's most north-eastern Heilongjiang area, traded with the Shang dynasty.

‘Cultural relics unearthed from these surrounding kingdoms share a commonality—all including Shang-style bronze containers,’ said Wang. ‘Although bronzes from surrounding kingdoms have their own characteristics, they were not as distinctive as Shu bronzes. The style of bronzes fully reflects the multiplex but unified characteristic of Chinese civilization.’

Archaeologists and anthropologists often compare civilizations of the same period, of different periods, and in different regions. ‘It appears surprising that the ancient Shu people would burn, smash, and bury such exquisite artefacts, but the phenomenon of fragmentation is quite common worldwide,’ commented Jing. For example, on the island of New Ireland in Papua New Guinea, the indigenous people still retain a sacrificial custom. To commemorate the dead, they make exquisite wood carvings, and then they smash them and burn them during a sacrificial ceremony.

Metallurgy, writing system and city have been considered the three defining elements of a civilization. However, in China three millennium ago, only the central Shang dynasty had oracle bone scripts; there is no existing evidence of a writing system in the ancient Shu and other surrounding kingdoms. According to Wang, ‘the absence of a writing system is supported by ancient documents. A civilization developed in a fixed region could effectively pass on through oral transmission without the need of written scripts. Even in the Shang dynasty of central China, only very few nobilities were able to use the oracle bone scripts.’

The three elements of civilization were posited on the basis of the ancient civilizations of Egypt and West Asia, although the Mayan civilization had no metallurgical technology. ‘Compared with metallurgy and writing, the emergence of royal power, countries, and cities, or the differentiation of social classes and social organizations, are more relevant criteria for defining a civilization. In fact, in recent years, with the ongoing discovery of more ancient civilizations, the international community is no longer so obsessed with these three elements. We are beginning to realize the diversity of civilizations,’ said Wang.

The Sanxingdui excavation programme on the six newly discovered pits will be completed within the next few years, with a complete archaeological report scheduled for 2025. Pits 7 and 8, from which no relics have been unearthed to date, are the two largest of the eight Sanxingdui pits discovered. What surprises do they have in store? Only time will tell.

